# Electronic Paper Displays in Hospital Operations: Proposal for Deployment and Implementation

**DOI:** 10.2196/30862

**Published:** 2021-08-04

**Authors:** Guruprasad D Jambaulikar, Andrew Marshall, Mohammad Adrian Hasdianda, Chenzhe Cao, Paul Chen, Steven Miyawaki, Christopher W Baugh, Haipeng Zhang, Jonathan McCabe, Jennifer Su, Adam B Landman, Peter Ray Chai

**Affiliations:** 1 Department of Emergency Medicine Brigham and Women's Hospital Boston, MA United States; 2 Department of Emergency Medicine Harvard Medical School Boston, MA United States; 3 Brigham Digital Innovation Hub Brigham and Women's Hospital Boston, MA United States; 4 Department of Psychosocial Oncology and Palliative Care Dana Farber Cancer Institute Boston, MA United States; 5 E Ink Corporation Billerica, MA United States; 6 Mass General Brigham Information Systems Somerville, MA United States; 7 The Koch Institute for Integrated Cancer Research Massachusetts Institute of Technology Cambridge, MA United States; 8 The Fenway Institute Boston, MA United States

**Keywords:** electronic ink, patient satisfaction, display systems, whiteboards, hospital, deployment, proposal, implementation, communication, engagement, efficiency, usage

## Abstract

**Background:**

Display signage is ubiquitous and essential in hospitals to serve several clerical, operational, and clinical functions, including displaying notices, providing directions, and presenting clinical information. These functions improve efficiency and patient engagement, reduce errors, and enhance the continuity of care. Over time, signage has evolved from analog approaches such as whiteboards and handwritten notices to digital displays such as liquid crystal displays, light emitting diodes, and, now, electronic ink displays. Electronic ink displays are paper-like displays that are not backlit and show content by aligning microencapsulated color beads in response to an applied electric current. Power is only required to generate content and not to retain it. These displays are very readable, with low eye strain; minimize the emission of blue light; require minimal power; and can be driven by several data sources, ranging from virtual servers to electronic health record systems. These attributes make adapting electronic ink displays to hospitals an ideal use case.

**Objective:**

In this paper, we aimed to outline the use of signage and displays in hospitals with a focus on electronic ink displays. We aimed to assess the advantages and limitations of using these displays in hospitals and outline the various public-facing and patient-facing applications of electronic ink displays. Finally, we aimed to discuss the technological considerations and an implementation framework that must be followed when adopting and deploying electronic ink displays.

**Methods:**

The public-facing applications of electronic ink displays include signage and way-finders, timetables for shared workspaces, and noticeboards and bulletin boards. The clinical display applications may be smaller form factors such as door signs or bedside cards. The larger, ≥40-inch form factors may be used within patient rooms or at clinical command centers as a digital whiteboard to display general information, patient and clinician information, and care plans. In all these applications, such displays could replace analog whiteboards, noticeboards, and even other digital screens.

**Results:**

We are conducting pilot research projects to delineate best use cases and practices in adopting electronic ink displays in clinical settings. This will entail liaising with key stakeholders, gathering objective logistical and feasibility data, and, ultimately, quantifying and describing the effect on clinical care and patient satisfaction.

**Conclusions:**

There are several use cases in a clinical setting that may lend themselves perfectly to electronic ink display use. The main considerations to be studied in this adoption are network connectivity, content management, privacy and security robustness, and detailed comparison with existing modalities. Electronic ink displays offer a superior opportunity to future-proof existing practices. There is a need for theoretical considerations and real-world testing to determine if the advantages outweigh the limitations of electronic ink displays.

## Introduction

### Background on Hospital-Based Signage

Display signage is ubiquitous in hospitals and is important for daily hospital operations and clinical care [1]. Signage serves a range of clerical, operational, and clinical functions, including waypoint finding; displaying directions, notices, and bulletins; and presenting and organizing clinical schedules. The use of signs in clinical care became standard practice after a 2001 report by the Institute of Medicine emphasized patient-centered care [2]. Clinicians demonstrated that whiteboards increased patient engagement, thereby increasing satisfaction, mitigating common medical errors, and enhancing continuity of care [3-5].

Over the past decade, plasma and liquid crystal display (LCD) televisions have replaced analog surfaces to convey information. These dynamic digital alternatives enable both public-facing and clinical display systems to refresh and display data longitudinally or continuously. Public-facing displays serve general needs, including providing directions and maps, bulletins, notices, and general information. These may require intermittent or continuous changes, depending on the purpose they serve. Displays serving as bulletin boards may require the ability to display multiple messages, either requiring significant space when presented as analog or requiring continuous refreshes when digitally presented. Clinical display systems present individualized patient data to clinicians or patients. These require more ad hoc changes when analog, creating a risk of inaccurate information due to lapses in manual updates. Alternatively, digital clinical displays lend themselves to a more automated approach through electronic health record (EHR) linkage. As the cost of digitizing display systems has decreased, there is renewed interest in replacing physical signage in hospital systems with a host of digital display options.

Electronic display screens have garnered attention as an effective alternative to traditional paper or printed signage. With increasing network integration, LCD and light emitting diode (LED) screens have demonstrated value despite their cost [6]. As part of a data network (ie, “internet of things”), electronic display screens are more autonomous, allowing hospitals to provide accurate real-time information without much, if any, human manipulation. Electronic display screens can be remotely updated from a central hub, reducing the time and manpower needed for maintenance. They may be more environmentally sustainable by reducing paper and chemical waste [6]. There has already been traction in adopting electronic displays for public-facing information such as hospital waypoint finding, announcements, event postings, public health messaging, and cafeteria menus. These screens also have the potential to replace standard dry-erase boards as an individualized clinical display, standardizing information that patients receive daily or, in some settings such as the clinic or emergency department, multiple times each day. Each hospital will face its own unique challenges regarding the cost of physical devices, energy use concerns, integration difficulties, and privacy concerns for displays of patient information. This can make adoption of patient-facing electronic displays difficult [7].

One potential alternative to traditional signage is the use of electronic ink displays. Electronic ink is made of microcapsules of black pigments and white pigments suspended in a clear fluid. When a positive or negative electric field is applied, corresponding particles move to the top of the microcapsule where they become visible to the viewer. These systems are low power, easy to read, and can be manufactured at scale. Commercially used in eReaders and store placards and, thus, already known to many consumers, the adoption of electronic ink displays may serve as a viable platform to deliver information in the hospital setting. In this paper, we described the use of electronic ink, a low-power, high-resolution display screen that can be adapted to multiple hospital uses. We presented several use cases for electronic ink displays and described an implementation schema for hospital systems seeking to explore the use of these displays both for public and clinical functions.

### Electronic Ink

**Figure 1:**
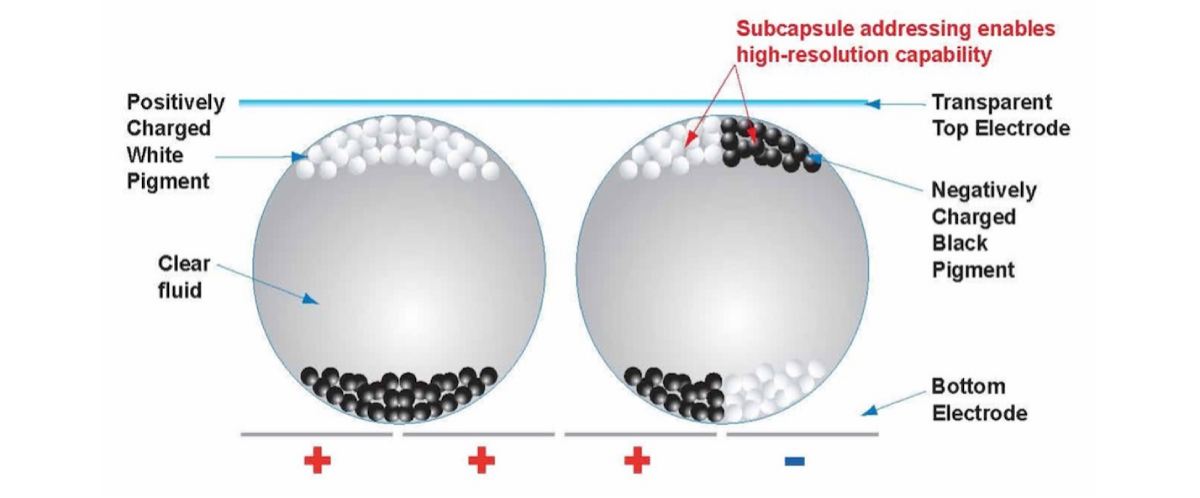
Working principle of electronic ink displays. Image provided by E Ink.

A formative development of paper-like electrophoretic surfaces was initiated at Xerox Palo Alto Research Center in 1974, under the pseudonym “Gyricon” [8,9]. A thin layer of transparent plastics with millions of small charged beads, akin to the dry powdery substances found in toner, rotates to present one colored side to the viewer when voltage is applied (Figure 1). The image, once set, does not change shape until new voltage patterns are applied.

In the mid-1990s, a professor at the Massachusetts Institute of Technology Media Lab tasked 2 of his students to take the early work of Gyricon and create a variant of electrophoretic ink that would allow for more precise image rendering and the ability to scale up to mass production. That project was commercialized in 1997 as E Ink [8]. Unlike Gyricon’s bichromal electrophoretic inks, E Ink instead uses microcapsules containing black and white pigments suspended in a clear fluid. These microcapsules are 50 microns in size and impart a more precise method of manipulating ink particles to create high-resolution, crisp displays. A core benefit of all electrophoretic inks is that they only need power to change an image, not to maintain it. This results in low-power displays in comparison with standard LCD panels (ie, 25-50 mW vs 100-200 mW for 1.5-2.5–inch displays) [10].

The end result is a readable, “paper-like” display that does not require backlighting and consumes significantly less power than typical LCD or LED screens. For the display of information that is static and does not require constant refreshes, electronic ink displays provide a potential alternative to more heavily powered LCD screens. This enables electronic ink technology to be used in places where a power source may not be available or constant backlighting is not required. Advances in both flexible screens and coloration enabled paper-like electronic ink screens to be applied to a variety of consumer facing signage such as grocery store signs, advertisements, and identification badges [11,12].

### Advantages of Electronic Ink Displays

There are several advantages to electronic ink displays compared with conventional LED or LCD screens. Because of the paper-like quality of electronic ink displays, they simulate analog paper surfaces and do not require backlighting, unlike conventional screens. This may make reading electronic ink displays easier by eliminating glare. Given the lack of backlighting, electronic ink displays emit no heat, decreasing the need for cooling systems or vents; this is a distinct advantage for display screens that are integrated into wall alcoves. Electronic ink displays are also lightweight and robust. Because they lack a liquid polymer or crystal layer and light source, the thickness and weight of electronic ink displays are markedly reduced. These features render them convenient to carry, transport, and install, and they are generally less susceptible to damage when dropped. This also allows electronic ink displays to be placed in locations where the weight and structural requirements of LCD panels may have been prohibitive.

Once the electronic ink has been set with the use of charges, it remains static until a refresh is triggered. This enables electronic ink displays to minimize power consumption, compared with other screen types [10]. Displays can, therefore, be powered over long periods of time using minimal power, even with conventional direct current batteries rather than requiring alternating-current wall plugs. Given the minimal power required to refresh a screen or display new information, electronic ink displays can be powered using power over ethernet. This allows for data to flow to the electronic ink display from a central controller while eliminating the need to use additional power infrastructures in existing hospital spaces. The end result for hospital groups is that, depending on the application, electronic ink displays may require less infrastructure support (eg, power over ethernet instead of requiring new electrical outlets), consume less energy, and emit little to no heat. These advantages may result in significant cost savings over the course of the device’s lifetime. Finally, each electronic ink display can be loaded with an integrated operating system. This provides flexibility to the platform and can eliminate the need to mirror the display of a tethered computer. By operating as standalone devices, electronic ink screens can be installed in a variety of locations and operational settings.

### Disadvantages of Electronic Ink Displays

There are several important limitations of electronic ink screens to consider before implementation in a clinical setting. First, due to a lower screen refresh rate compared with LED or LCD screens, electronic ink displays are not ideal for displaying video, animations, or rapidly changing information. Because of a set number of ink pigments suspended in electronic ink, colors are also limited, compared with the wide palette of colors available in LCD and LED displays. However, recent advances in microencapsulation have enabled combinations of colors with white pigment to enable color electronic ink displays. With continued development, future iterations of these systems may produce different colors that advance the available color palette for electronic ink displays, such as the four-pigment system, Advanced Color ePaper, that is in development at E Ink [13].

## Methods

### Potential Applications in Hospital Operations

Numerous potential applications for electronic ink displays exist in a hospital setting. We classify applications into 2 categories: public-facing displays and clinical display systems (Table 1).

**Table 1:** Potential applications of electronic ink displays to improve hospital operations.

Criteria	Type of display	
	Public-facing displays	Clinical displays
Potential applications	Notices and bulletins for employees, patients, and caregivers Way-finders and maps Location tags and placards Guidelines and regulations Public education and awareness messages	Door signs and bedside cards Digital communication boards in patient rooms Tablet devices with access to medical records

### Public-Facing Applications

Signage can be used to provide wayfinding and information to visitors in the hospital setting. Hospital settings are often complex and growing and can encompass several buildings built in different eras, creating wayfinding challenges. Easy-to-follow signage and legible directions are key to reducing stress and improving satisfaction for patients when navigating these campuses. Shared clinical spaces, offices, and conference rooms may be used for different purposes at different times and require frequent updates to avoid confusion. Electronic ink displays offer the opportunity to replace some of these screens, providing easily understandable signage that can be updated from a central location on demand.

Portable, lightweight electronic ink displays can also be used instead of noticeboards. Often pinboards and television screens are used to display bulletins and general information to visitors. If linked to a centralized location on the local network, updating electronic ink displays require less manual work than these traditional signage methods. They are potentially lower in total ownership cost and more energy efficient than LED and LCD screens for this purpose, and installation may be easier, especially, for locations that do not require intensive refreshing of screens. Since they are able to be attached to mobile carts, they are more portable, making it less challenging to transport them across different indoor or outdoor locations around the hospital. During the COVID-19 pandemic, signage has been used to provide reminders to hospital staff and visitors of important public health measures such as social distancing, hand hygiene, and mask wearing. The rapid pace at which guidelines continue to change around public health measures, testing requirements, and other COVID-19–related interventions suggests that the use of electronic ink displays could be an effective, low-energy method to provide up-to-date information to hospital visitors.

### Patient-Facing Applications

**Figure 2:**
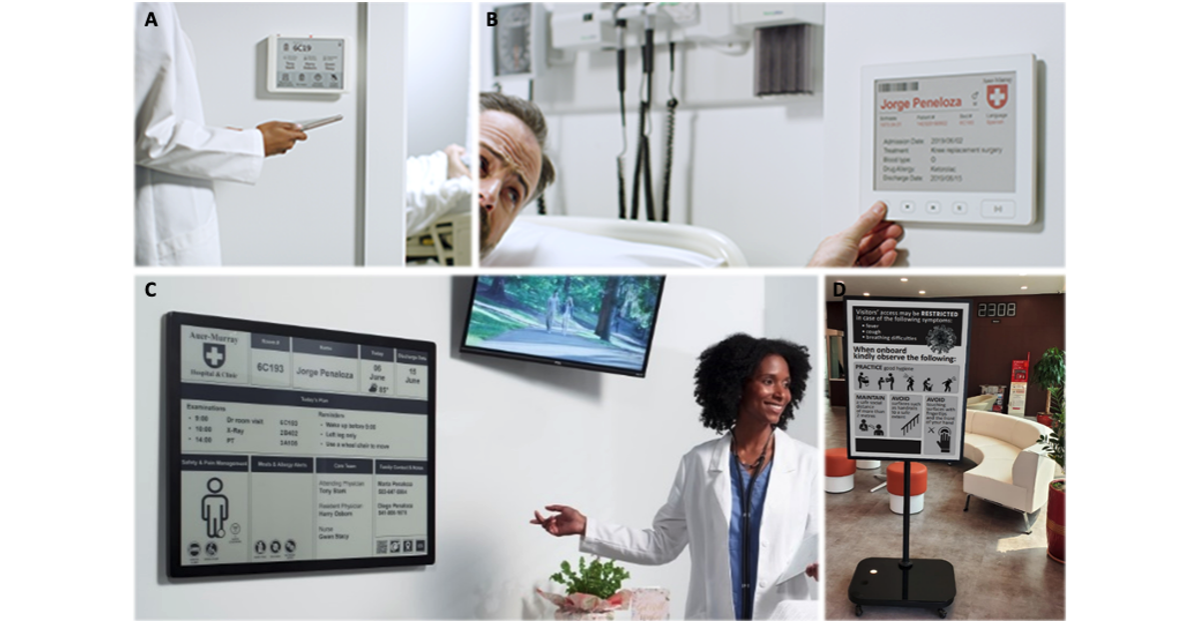
Diverse applications using electronic ink displays in the health care setting. (A) Door sign. (B) Bedside card. (C) Patient room information board. (D) Visitor signage. Images provided by E Ink.

Electronic ink also has utility for clinical displays. In their simplest form, electronic ink displays can be used as a door sign or bedside card, available in several sizes and resolutions (Figure 2). They are easily installed with standard wall anchors, or they can be hung on a hospital bed; they are powered for several months with AA, AAA, or rechargeable batteries. These small electronic ink displays can contain information about patients, including their location or next steps in their plan (eg, travel to radiology department for an x-ray or a cardiac stress test). When integrated with radiofrequency identification or low-energy Bluetooth beacons, electronic ink displays can also function as a wayfinding application. These applications may improve efficiency, help provide on-demand data about a patient’s itinerary, and relocate on-demand changes in scheduling or clinical information to the bedside. Digital door signs and bedside cards can be programmed to display as little or as much information as desired and can also be used to display patient safety elements such as precautions, allergies, and alerts.

**Figure 3:**
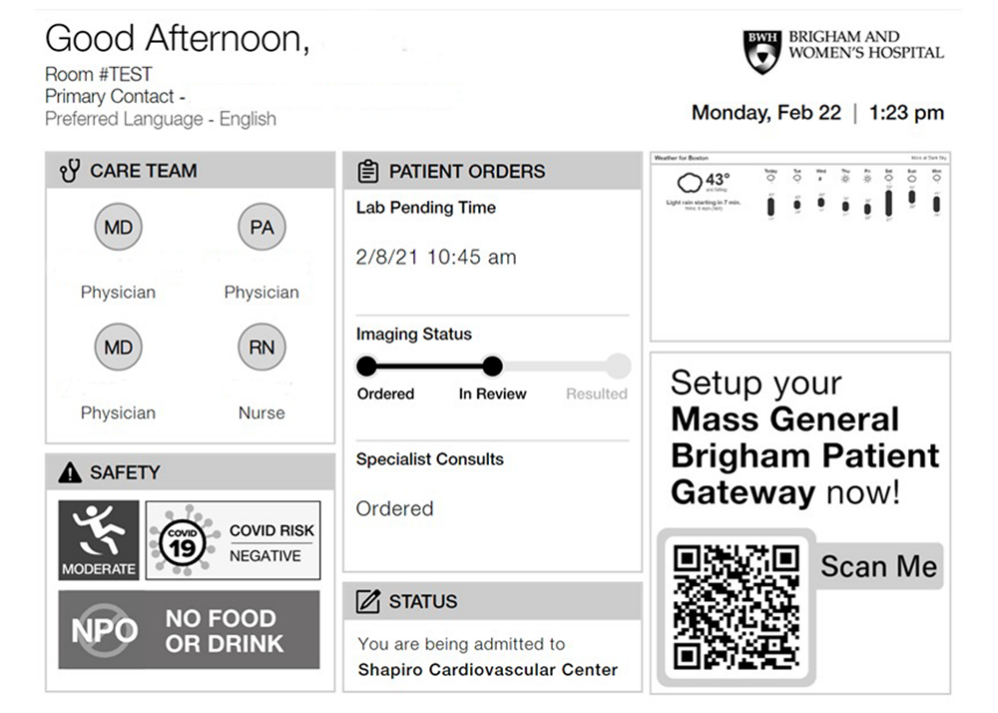
Electronic ink communication board. NPO: nothing by mouth.

Dry-erase boards have been used in the hospital to orient patients to the date, provide information regarding their care team members, and even communicate pain scores [14-16]. In addition, paper and laminated signs have been used outside of patient rooms to convey information regarding isolation status, fall risk, and need for personal protective equipment [4]. In some instances, hospitals display daily plans to inpatients and their families, and, for acutely ill patients in the emergency department, whiteboards can be used by the teams to coordinate care. A significant drawback to the use of paper or traditional whiteboards is the need to manually and consistently update them at set intervals. In busy hospital settings, this can result in outdated or inaccurate information, creating new safety risks that the tool was originally intended to mitigate. As an alternative to either a whiteboard or a backlit screen such as a television screen, electronic ink screens can be used to display patient information (Figure 3). Since electronic ink screens do not emit light, they are less distracting to patients who are trying to sleep or rest during their stay. This may be instrumental in preventing delirium caused by persistent light stimuli from LCD screens. By providing an indirect stimulus concerning the course of their clinical care, it is possible that electronic ink displays can help provide orientation to patients with prolonged stays in the hospital, thereby addressing disorientation and delirium [17,18]. Patients are becoming increasingly more comfortable with the use of technology to enhance their care. In a 2012 survey study of an urban emergency department, approximately 90% of patients preferred technology-based behavioral interventions [19]. Communication boards can be configured to display data customized for each unique clinical environment. Basic functions include displaying information to orient patients, such as date, time, and names and roles of the current clinical team. Information on diagnostic tests and imaging, as well as final disposition, may help guide patients and their families to understand their clinical course and anticipate potential events that may happen in the hospital. For patient-facing screens, additional functionality may include displaying local weather, transit schedules, cafeteria menus, and important hospital notices, thereby providing on-demand information to patients and their families. Strong communication and efficient information delivery have been associated with improved patient satisfaction, which may, ultimately, influence hospital choice and improve quality of care [20,21].

The success of electronic ink displays in hospitals is contingent on their integration with existing information systems. Legislative and regulatory changes have promoted significant advances in interoperability. These advances have created standard methods for connecting applications to exposed application programing interfaces (APIs) in EHRs, allowing for seamless data exchange. Using this exchange, electronic ink devices may be configured to display real-time information directly from EHRs. Additional discussion on protocols to directly exchange information can be found under the subheading “Technological Considerations” in the Discussion section of this paper.

## Results

We are planning several pilot studies to evaluate the use of electronic ink screens in various hospital settings. We hope to use these pilot studies to help delineate the best practices and use cases for electronic ink screens in clinical care and hospital operations. These pilot studies will also help develop the information security infrastructure and support needed to manage multiple electronic ink screens at the same time. This programming architecture also permits custom displays of different data on various screens. At this time, electronic ink displays are deployed in our emergency department to display information about a patient’s emergency stay, and we are investigating their effect on patient satisfaction. Other use cases will include wayfinding through smaller display screens affixed to hospital beds, two-way asynchronous communication between patients and clinicians through the electronic ink screens, and patient identification in the operating room.

## Discussion

### Technological Considerations

#### Connectivity

Ensuring secure and reliable network connectivity is an important consideration in electronic ink display deployments. Electronic ink displays can be deployed in several different manners. The simplest manner is with limited network connectivity, requiring manual entry of information to be displayed. One way to deploy this is to create a virtual environment—a server-based command control for network displays—on the hospital network, which is directly connected to the display. Eventually, the most sustainable and future-proof method will be to connect these displays to data sources that are configured to update specified information such as EHRs automatically and as close to real time as possible. This would add the most value to the efficiency and accuracy of clinical displays inside and outside patient rooms.

Deploying a large number of electronic ink displays in a hospital environment has different considerations compared with the implementation of LCD display screens. Unlike LCD screens that require a persistent internet connection to display information, electronic ink screens only require data connection to change the information displays. Depending on the intended information, electronic ink screens may, therefore, require less internet bandwidth compared with LCD screens. The nature of the intermittent data of electronic ink displays may also pose less of a network security risk, as there is no maintained continuous internet connection; instead, the central architecture pings each electronic ink display only when new data are displayed. Until network infrastructure can handle the traffic, the use of dedicated networks in the form of virtual machines may help ensure efficient display performance and also minimize the chance of unintended disruptions to the primary hospital network.

#### Privacy and Security

When considering the use of electronic ink displays, hospital systems should understand the potential privacy and security risks. Privacy breaches can occur when unencrypted data that contain protected health information (PHI) are intercepted or when the incorrect data are transmitted to an electronic ink display. For example, manual updates of electronic ink displays in patient rooms could inadvertently expose other individuals in the room to PHI, or, if a patient’s information is displayed to the incorrect screen, this could result in a privacy violation. Patient privacy is a prime concern when PHI is transmitted to displays from EHRs. To protect against breaches in patient privacy, data transmission should avoid use of PHI or use the minimum PHI necessary for care delivery. PHI or other sensitive information that needs to be transmitted should use modern encryption protocols. Given that electronic ink displays are visible to anyone in the physical room, masking PHI on the screen could also be considered, such as displaying patient initials instead of full names.

Despite best practices, there remains the possibility that manually entered data in an electronic ink display could inadvertently be erroneous or expose PHI to another patient if an incorrect display is loaded by mistake. These errors are significantly mitigated through the use of automated checks and integrated rules in the electronic medical record that may prevent the display of data in inappropriate locations. Notwithstanding these technical mitigation strategies, we recommend additional physical mitigation, including a physical shut-off button to wipe the screen in case individuals find unauthorized information displayed on the screen.

Network security can be compromised when smart displays are used as an entry point to hospital networks or used as a distributed denial of service attack. Understanding these risks and creating strategies to effectively mitigate them are central to safely deploying these technologies in hospitals (Table 2).

**Table 2:** Privacy and security considerations for electronic ink displays.

Potential concern	Potential solutions
Privacy and data breaches	Communicate deidentified data where possible Suspend continuous network connection and data transfer when not required Enable electronic ink displays to communicate via independent networks to minimize integration with hospital networks unless necessary Limit network access by only delegating accounts that need access and following institutional password requirements Establish a log system to audit and document evidence of undesired activity Develop a risk-mitigation plan and an incident-response policy that may be implemented in case of emergency
Distributed denial of service attacks	Establish a log system to audit and document evidence of undesired activity Displays placed on separate networks with intermittent limited access to hospital servers only as required Ensure server updates and vulnerabilities are addressed
Failure	Staggered adoption with careful testing of failure rates Initial use in conjunction with existing standard practices Develop a risk-mitigation plan and an incident-response policy that may be implemented in case of emergency

Open firewall ports, used to deliver data to screens, may provide a portal to enter a hospital network and interdict critical health information or conduct malicious attacks against hospital infrastructure [22]. To alleviate these risks, smart display systems can be programmed to only connect to a hospital network for a brief period, during which refreshes are performed and data are transmitted. As is best practice, smart devices should be isolated from main hospital networks.

In order to transmit sensitive notifications containing protected health information, electronic ink displays should securely connect to encrypted wireless networks using Wi-Fi Protected Access 2 connection. Wi-Fi Protected Access-enterprise encryption systems, which require a user to enter a unique username and password to log into the network, provide an even higher layer of security necessary for networks that transmit confidential information. This way, even if a hacker learns the password of one device, they cannot compromise the entire system. Another option is to use two-factor authentication to validate the administrator who is accessing the configuration of the electronic ink screen. This additional layer of security may help mitigate bot-based hacking attempts.

Malicious users with control over smart devices could also conduct distributed denial of service attacks by sending rapid triggers from the devices, in an attempt to overwhelm the hospital network [23,24]. As noted previously, limiting continuity of network transmission should address this issue. Finally, good stewardship of smart devices is paramount to protecting security, such as limiting access only to highly trained staff and continually encouraging good practices.

#### Systems Integration

The success of electronic ink devices in the hospital relies heavily on the ability to effectively update the information displayed from a central location. For public-facing signage, this likely requires the establishment of a centralized dashboard for content management. Patient-facing displays, on the other hand, likely require integration of EHR for maximum efficiency.

Improvements in interoperability and a nationwide focus on providing patients access to their entire medical records have made it feasible to propose patient-facing applications that integrate EHRs directly. As a part of the Meaningful Use Act [25], hospitals were incentivized to provide access to health care information to patients. The more recent information-blocking provision of the 21st Century Cures Act [26] removed nearly all barriers to patients’ ability to access their hospital records. Electronic ink displays may act as a vehicle to provide on-demand access to pertinent patient information that could reduce barriers to accessing health records as well as assisting hospitals in satisfying the requirements of the Meaningful Use Act [27]. For example, emergency department patients can obtain their personal laboratory results or understand a status update from a consultant who has been asked to evaluate them.

Exchange of clinical information between electronic ink devices and EHRs should be built around widely accepted standards. Fast Healthcare Interoperability Resources [28] provides an open-source and widely accepted means for packaging and delivering clinical data. Although some form of health information exchange is required as a part of the Meaningful Use Act, each institution's API may differ. The ideal systems integration uses the APIs health level 7 or Fast Healthcare Interoperability Resources, enabling and automating information exchange on electronic ink screens [29].

### A Framework for Electronic Ink Display Deployment

**Figure 4:**
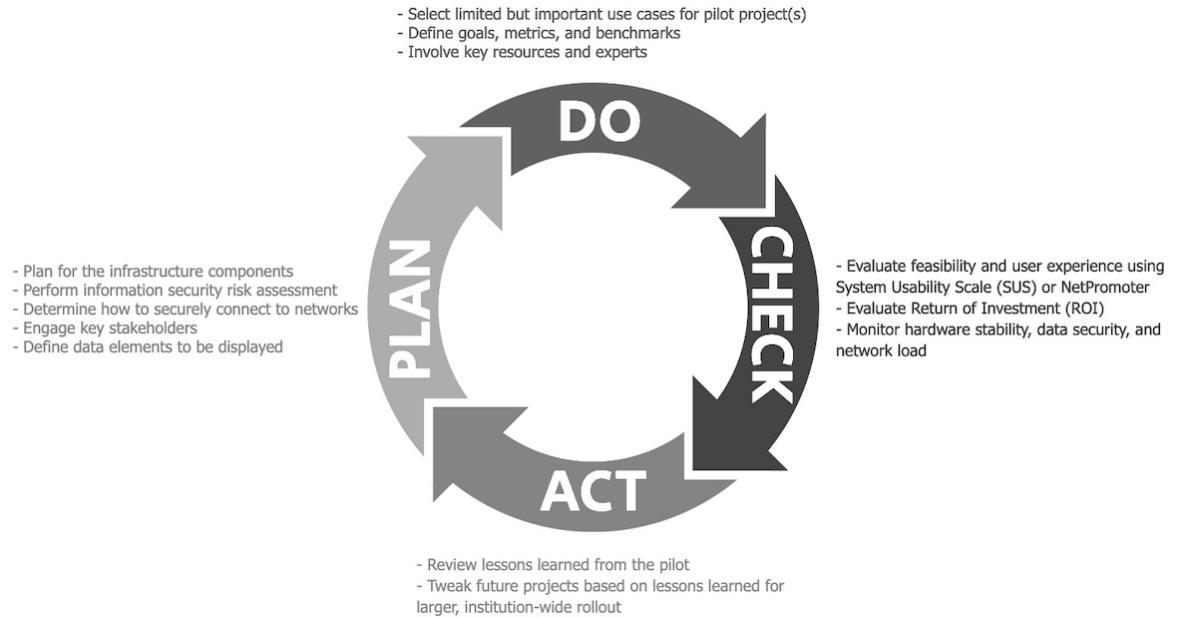
A proposed framework to deploy and evaluate the impact of electronic ink displays in a hospital setting.

For health care organizations that seek to deploy electronic ink displays, we recommend steps grounded in the plan-do-study-act (PDSA) method (Figure 4) [30]. The PDSA method is often used to accelerate quality improvement initiatives by rapidly testing changes through structured planning, implementation, observation, and iterative improvements based on pilot studies [30]. For successful deployment of a novel technology, it may be helpful to form a centralized committee of network specialists, hospital administration, and clinical experts who understand outcomes surrounding the use of electronic ink displays and information security, to ensure that all stakeholders required to successfully deploy an electronic ink display can assemble and map key tasks prior to implementation. Establishing a central data server that can query and modify displays in a secure manner will be critical to the success of pilot studies, particularly, for electronic ink displays, and should be developed prior to deployment of electronic ink displays in the clinical setting.

As part of the “plan” phase, the first step in an electronic ink display implementation is to plan for the infrastructure components. Since the displays will require network connectivity, hospital information security officers should be engaged to perform a risk assessment of the technology and mitigate any high-priority risks identified. It is also important to limit the initial scope of work for any display during the process of deployment. This permits a gradual rollout of electronic ink displays and allows adequate space for implementation issues to be resolved by the study team. Several key stakeholders, including clinical and nonclinical staff, patients, and patient advocates, should discuss and finalize the data elements that make the most sense in a given scenario. Key discussion points should center around important identifying information that may be displayed on the screen.

As part of the “do” phase, the team should select limited, yet important, use cases that would benefit from a brief pilot run. Benchmarks for success of the pilot study and the duration of the study should be established in advance, to provide clear parameters and expectations around deployment. Despite selecting individual pilot studies, a central resource of information technology and device and programming expertise should help govern and manage electronic ink displays. This will ensure that there is continuity around different projects and that data from projects remain unified in a central location. Additionally, this mechanism will allow for seamless transfer of operating systems and platforms to additional investigations planned by the study team.

As part of the “study” phase, the pilot study should be evaluated to assess feasibility, return on investment, and user experience of the electronic ink displays. Investigators may consider the use of validated measures such as the System Usability Scale or Net Promoter score to understand usability, acceptability, and satisfaction associated with implementation of the system [31-34]. In addition, hardware stability, power consumption and cost, data security and quality, and network load should be monitored during the pilot study.

As part of the “act” phase, lessons learned from the pilot study, including technical, workflow, and other components of the socio-technical model for health information technology, should be carefully reviewed [35]. Based on the pilot studies completed, a use case for widespread implementation should be developed. Messaging with and training of the staff who will operate and interact with electronic ink displays should occur prior to the date when the displays become activated. To prepare for larger rollouts, processes and protocols should be adjusted based on these lessons learned. A dedicated governance process team, including the project team, information technology staff, and institutional leaders, is essential for identifying the appropriateness of employing smart displays for any use case. It is also important to understand the reliability of the technology and the impact it has on technical infrastructures, so it can be safely used for its desired tasks.

### Conclusions

Electronic ink displays may be a valuable tool to help optimize hospital operations and communications. They have a variety of use cases for both patients and staff. Key technical considerations for a successful deployment include an appropriate network connectivity, a robust content management process, and a careful configuration that minimizes privacy and security risks. The PDSA framework may guide hospitals, starting with a small pilot study and iteratively refining the process until eventually scaling to the entire organization. Electronic ink displays present a tremendous opportunity to future-proof existing analog processes while overcoming some of the disadvantages of commonly used digital modalities. The promise might outweigh the minor limitations, but there is still a need for testing in real-world health care environments to rigorously evaluate and determine the impacts of electronic ink displays.

## Abbreviations

API: application programing interface
EHR: electronic health record
LCD: liquid crystal display
LED: light emitting diode
PDSA: plan-do-study-act
PHI: protected health information
